# Supplementary material for: Exploring Aeromonas veronii in Migratory Mute Swans (Cygnus olor): A Debut Report and Genetic Characterization
Source: Vet Sci. 2025 Feb 13;12(2):164. doi: 10.3390/vetsci12020164 (PMC11861175; doi:10.3390/vetsci12020164)
Supplement: Supplementary file 1 [file vetsci-12-00164-s001.zip › vetsci-3421058-supplementary.pdf]

Tables

Table S1. Primers used in this study for detection of the viral pathogen.

Primers	Sequences (5'-3')	Amplicon size (bp)	Reference
AIV-F	GGCGACTACTACCAACCCA	435	Yao et al., 2019
AIV-R	CTGCTGTTCCTGCCGATAT		
GRV-F	TGAGACGCCTGACTACGATT	380	Niu et al., 2017
GRV-R	ATGCTTGGAGTGAGACGACT		
TMUV-F	GCCACGGAATTAGCGGTTGT	401	Su et al., 2011
TMUV-R	TAATCCTCCATCTCAGCGGTGTAG		
GPV-F	AGACTTATCAACAACCATCAYT	779	Li et al., 2017
GPV-R	TCACTTATTCCTGCTGTAG		
GHPV-F	GAGGTTGTTGGAGTGACCACAATG	144	Zhang et al., 2018
GHPV-R	ACAACCCTGCAATTCCAAGGGTTC		

References

M. Yao, X. Y. Zhang, Y. F. Gao, S. Q. Song, D. N. Xu,L. P. Yan, Development and application of multiplex PCR method for simultaneous detection of seven viruses in ducks, BMC Vet Res. 15 (1) (2019) 103.

Niu X, Zhang B, Yu X, Zhang X, Dou Y, Tang Y, Diao Y. Preparation and evaluation of goose reovirus inactivated vaccine. BMC Vet Res. 2017 Jul 6;13(1):214.

J. L. Su, S. Li, X. D. Hu, X. L. Yu, Y. Y. Wang, P. P. Liu, X. S. Lu, G. Z. Zhang, X. Y. Hu, D. Liu, X. X. Li, W. L. Su, H. Lu, N. S. Mok, P. Y. Wang, M. Wang, K. G. Tian,G. F. Gao, Duck egg-drop syndrome caused by BYD virus, a new Tembusu-related flavivirus, PLoS One. 6 (3) (2011) e18106.

P. Li, R. Zhang, J. Chen, D. Sun, J. Lan, S. Lin, S. Song, Z. Xie,S. Jiang, Development of a duplex semi-nested PCR assay for detection of classical goose parvovirus and novel goose parvovirus-related virus in sick or dead ducks with short beak and dwarfism syndrome, Journal of virological methods 249 (2017) 165-169.

Table S2. Primers and PCR conditions[a] for virulent factors.

Gene	Virulent factors	Primer sequences (5' to 3')	Annealing temperature (ºC)	Amplicon size (bp)	Reference
hlyA	Hemolysin A	F:GGCCGGTGGCCCGAAGATACGGG<br>R:GGCGGCGCCGGACGAGACGGG	62	597	Wong et al. 1998
alt	Heat-labile cytotonic enterotoxin	F:TGACCCAGTCCTGGCACGGC<br>R:GGTGATCGATCACCACCAGC	63	442	
act	Cytotoxic enterotoxin	F:AGAAGGTGACCACCACCAAGAACA<br>R:AACTGACATCGGCCTTGAACTC	64	232	
ast	Heat-stable cytotonic enterotoxin	F:TCTCCATGCTTCCCTTCCACT<br>R:GTGTAGGGATTGAAGAAGCCG	63	331	
aerA	Aerolysin	F:CCTATGGCCTGAGCGAGAAG<br>R:CCAGTTCCAGTCCCACCACT	63	431	Nawaz et al. 2010
fla	Flagella	F:TCCAACCGTYTGACCTC<br>R:GMYTGGTTGCGRATGGT<br>R:GGCAGGTTGAACAGCAGTATCT<br>R:GGCTCATGCGTAACTCTGGT<br>R:CGCTGGTGTTGGCCAGCAGG	55	608	
ela	Elastase	F:ACACGGTCAAGGAGATCAAC<br>R:CGCTGGTGTTGGCCAGCAGG	59	513	
lip	Lipase	F:CAYCTGGTKCCGCTCAAG<br>R:GTRCCGAACCAGTCGGAGAA<br>R:GCCAAGCTTACTTAAATTGATTCAC TCAGC	62	382	Sen and Rodgers 2004

[a] PCR thermocycle conditions for each reaction is as follows, initial denaturation of 94 ºC for 5 min followed by a total of 35 cycles of amplification. Each cycle consisted of 94 ºC denaturation for 30 s, annealing for 50 s and 72 ºC extension for 1 min, and a final extension for 7 min at 72 ºC.

References

Nawaz, M., Khan, S.A., Khan, A.A., Sung, K., Tran, Q., Kerdahi, K., Steele, R., 2010. Detection and characterization of virulence genes and integrons in *Aeromonas veronii* isolated from catfish. Food microbiol. 27, 327–331. https://doi.org/10.1016/j.fm.2009.11.007.

Sen, K., Rodgers, M., 2004. Distribution of six virulence factors in *Aeromonas* species isolated from US drinking water utilities: a PCR identification. J Appl. Microbiol. 97, 1077–1086. https://doi.org/10.1111/j.1365-2672.2004.02398.x.

Wong, C., Heuzenroeder, M.W., Flower, R. 1998. Inactivation of two haemolytic toxin genes in *Aeromonas hydrophila* attenuates virulence in a suckling mouse model. Microbiology. 144, 291–298. https://doi.org/10.1099/00221287-144-2-291.

Table S3. Antimicrobial resistance profiles of *Aeromonas veronii* HNZZ-1/2022.

Antimicrobial Class	Antimicrobial Agent	Interpretive Categories and MIC Breakpoints (μg/mL)			MIC (μg/mL)	Susceptibility
		S	I	R		
Carbapenems	Meropenem	≤1	2	≥4	32	R
Oxazolidinones	Linezolid	≤2	-[a]	-	32	-
Penicillins	Ampicillin	≤8/4	16/8	≥32/16	32	R
Fluoroquinolones	Enrofloxacin	≤0.25	-	≥0.5	0.625	R
Cephems	Cefoxitin	≤8	16	≥32	8	S
	Ceftazidime	≤4	8	≥16	8	I
Aminoglycosides	Gentamicin	≤4	8	≥16	2.5	S
Tetracyclines	Doxycycline	-	-	-	0.125	-
	Tigecycline	-	-	-	0.125	-
Polymyxin	Colistin	-	-	-	8	-
Chloramphenicols	Florfenicol	-	-	-	0.25	-
Pleuromutilin	Tiamulin	-	-	-	128	-
Aminocyclitols	Spectinomycin	-	-	-	16	-
Fosfomycins	Fosfomycin	-	-	-	64	-

Interpretative break points: based on CLSI guideline M100-30th ed (2020); S-susceptible, I-intermediate, R-resistant.

[a] Not determinable.

Figures

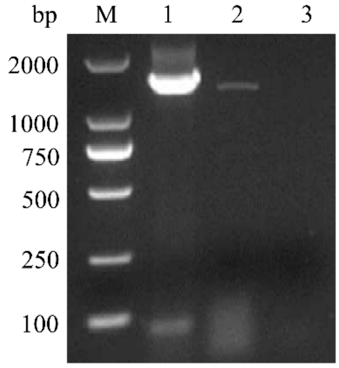

Figure S1. Agarose gel electrophoresis of PCR products of the 16S *rRNA* and *gyrB* gene of HNZZ-1-2022. M. DL2,000 DNA Marker; 1. 16S *rRNA* gene; 2. *gyrB* gene; 3. Negative control.
